# Cerebral Science: Studies in Anatomical Psychology

**Published:** 1901-09

**Authors:** 


**Cerebral Science: Studies in Anatomical Psychology.** By WALLACE WOOD, M.D. Pp. 128. London: Baillière, Tindall and Cox. 1901.

The author endeavours to show that we must study the brains of animals if we are to arrive at a knowledge of the composition of human nature, and in his preface he indicates the chief aim of the book, which culminates in "Ethology: *how to regenerate man*, *how to make the better man and the better woman*, *how to build the new body*, *the new brain*, *and the new city*, how to shape the new world. . . ." The *hautes études* for the new century are astronomy, botany, aerostation, and cerebrology: there is enough to do—'*Laboremus*.'

Further (p. 22), we are told that "the seven wonders of the cerebral world are the mental jaws of the tiger, the mental ear of the cat, the mental eye of the ape, the anthemion of the ox, and in man the high front, the speaking lobe, and the Rolando lobe." The views of the author are too fantastic for any analysis and too condensed for any epitome; but the book contains most original ideas and suggestions which will set other gyri into unwonted activity in new realms of characterology, cerebrology, gyrology, psychometrology, and so on.

The author's psycho-dynamo goes much further in the direction of psychical localization than has been dreamt of by Ferrier; and the volume concludes with a comparison of the herbivorous, the carnivorous, and the omnivorous order of brain—say, the type taurus, the type leo, and the type homo.

To the primate the Maker "has given light, mildness, and dexterity; and to the magnate, with these as a foundation, He has added the lobules of the agape and the nous, the eidos and the logos." We have scarcely yet learnt even the alphabet of this new science.

